# Mediation of 6‐year mid‐childhood follow‐up outcomes after pre‐school social communication (PACT) therapy for autistic children: randomised controlled trial

**DOI:** 10.1111/jcpp.13798

**Published:** 2023-04-24

**Authors:** Sophie Carruthers, Andrew Pickles, Tony Charman, Helen McConachie, Ann Le Couteur, Vicky Slonims, Patricia Howlin, Rachel Collum, Erica Salomone, Hannah Tobin, Isobel Gammer, Jessica Maxwell, Catherine Aldred, Jeremy Parr, Kathy Leadbitter, Jonathan Green

**Affiliations:** ^1^ Department of Psychology Institute of Psychiatry, Psychology & Neuroscience, King's College London London UK; ^2^ Department of Biostatistics and Health Informatics Institute of Psychiatry, Psychology & Neuroscience, King's College London London UK; ^3^ Population Health Sciences Institute Newcastle University Newcastle upon Tyne UK; ^4^ Evelina London Children's Hospital London UK; ^5^ Department of Psychology University of Milano‐Bicocca Milan Italy; ^6^ University of Manchester Manchester UK; ^7^ Manchester Academic Health Sciences Centre, Royal Manchester Children's Hospital University of Manchester Manchester UK

**Keywords:** Autism spectrum disorder, developmental psychopathology, mediation, early intervention, structural equation modelling

## Abstract

**Background:**

There are very few mechanistic studies of the long‐term impact of psychosocial interventions in childhood. The parent‐mediated Paediatric Autism Communication Therapy (PACT) RCT showed sustained effects on autistic child outcomes from pre‐school to mid‐childhood. We investigated the mechanism by which the PACT intervention achieved these effects.

**Methods:**

Of 152 children randomised to receive PACT or treatment as usual between 2 and 5 years of age, 121 (79.6%) were followed 5–6 years after the endpoint at a mean age of 10.5 years. Assessors, blind to the intervention group, measured Autism Diagnostic Observation Scale Calibrated Severity Score (ADOS CSS) for child autistic behaviours and Teacher Vineland (TVABS) for adaptive behaviour in school. Hypothesised mediators were child communication initiations with caregivers in a standard play observation (Dyadic Communication Measure for Autism, DCMA). Hypothesised moderators of mediation were baseline child non‐verbal age equivalent scores (AE), communication and symbolic development (CSBS) and ‘insistence on sameness’ (IS). Structural equation modelling was used in a repeated measures mediation design.

**Results:**

Good model fits were obtained. The treatment effect on child dyadic initiation with the caregiver was sustained through the follow‐up period. Increased child initiation at treatment midpoint mediated the majority (73%) of the treatment effect on follow‐up ADOS CSS. A combination of partial mediation from midpoint child initiations and the direct effect of treatment also contributed to a near‐significant total effect on follow‐up TVABS. No moderation of this mediation was found for AE, CSBS or IS.

**Conclusions:**

Early sustained increase in an autistic child's communication initiation with their caregiver is largely responsible for the long‐term effects from PACT therapy on autistic and adaptive behaviour outcomes. This supports the theoretical logic model of PACT therapy but also illuminates fundamental causal processes of social and adaptive development in autism over time: early social engagement in autism can be improved and this can have long‐term generalised outcome effects.

## Introduction

There is good evidence from randomised controlled trials (RCTs) that aspects of social communication and other relevant outcomes can be improved for young autistic children through Developmental Social Communication (DSC) and Naturalistic Developmental Behavioural Interventions (NDBI) approaches (Crank et al., 2021; French & Kennedy, 2018; Green & Garg, 2018; Sandbank et al., 2020). Such approaches are characterised by targeting developmentally meaningful processes in autism development, such as dyadic social communication, social motivation and joint attention and joint engagement, all thought to have downstream effects on social development, communication and broader development (Charman, 2003; Mundy, Sigman, & Kasari, 1990; Siller & Sigman, 2002, 2008). The general pattern of findings is for consistent moderate to good effects on targeted outcomes close to the intervention context, such as dyadic initiations of communication or joint engagement, but much less evidence of treatment effect on more ‘distal’ child development outcomes beyond the intervention, such as language, autism social communication and other symptoms (Crank et al., 2021; Green & Garg, 2018; Sandbank et al., 2020). The quality of trial methodology and reporting in these studies is variable and reported intervention effects reduce considerably when potentially biased caregiver‐reported outcomes are excluded, however, effects do remain from some trials with blinded outcomes (Sandbank et al., 2020). Despite the growing numbers of RCTs testing interventions for autistic children, very few follow‐up studies have been conducted, conceptually important for an intervention in a developmental condition. Those that have been done reported mixed findings, but there is evidence that both proximal dyadic (Kaale, Fagerland, Martinsen, & Smith, 2014; Poslawsky et al., 2015) and distal child outcomes (Estes et al., 2015; Green & Wan, 2017; Gulsrud, Hellemann, Freeman, & Kasari, 2014; Kasari, Gulsrud, Freeman, Paparella, & Hellemann, 2012; Pickles et al., 2016; Whitehouse et al., 2021) can be found up to 2 years or so following therapy. To date, only two studies have investigated longer‐term outcomes, for instance into mid‐childhood following early pre‐school intervention (Gulsrud et al., 2014; Pickles et al., 2016).

Some DSC and NDBI interventions have focused on naturalistic parent‐mediated approaches, aiming to increase parents' use of synchronous, responsive and non‐directive interaction styles (Green & Garg, 2018; Nevill, Lecavalier, & Stratis, 2018). Such changes in parent interaction style can mediate improved dyadic parent–child engagement in some studies (Gulsrud, Hellemann, Shire, & Kasari, 2016; Shih, Shire, Chang, & Kasari, 2021) and both proximal and distal child outcomes in others (Aldred, Green, Emsley, & McConachie, 2012; Pickles et al., 2015; Watson et al., 2017). Paediatric Autism Communication Therapy (PACT) is a parent‐mediated intervention that uses focused video feedback techniques to increase parents' awareness, understanding and synchronous dyadic response with their autistic child (synchronous interaction being that which gives accurate timely response to child behaviour and communication, maintaining its flow; Siller & Sigman, 2008). General developmental theory suggests that the child may respond to this with increased dyadic social engagement, and communication initiation, which may itself then generalise into improve social functioning in other contexts and over time. No direct therapeutic work is done by the therapist with the child in PACT intervention, and no behaviour‐learning methods are used to try to alter the child behaviours. The most substantial RCT of PACT to date tested the 13‐month intervention plus treatment as usual (TAU), compared to TAU alone. It showed large intervention effects to increase parent synchrony and significant effects to increase child dyadic communication initiations with the parent at both midpoint (TAU *n* = 72, 0.28 (*SD* 0.19); PACT *n* = 74, 0.40 (*SD* 0.22); see Pickles et al., 2015, 2016, figure 2) and 13‐month endpoint (Green et al., 2010). Distal endpoint outcome using the researcher Autism Diagnostic Observation Schedule (ADOS) showed trends on both child social communication (SC, the original primary outcome) and repetitive restricted (RRB) symptom domains which, when analysed together as an overall symptom measure, were significant in effect on both ADOS Calibrated Severity Score (CSS) and ADOS‐2 total algorithm score (Carruthers et al., 2021; Pickles et al., 2016).

Mediation analysis of this initial 13‐month trial period evidenced support for the proposed intervention mechanism (Pickles et al., 2015). The mediation model attributed almost all of the endpoint changes in ADOS social communication outcomes to change in child's dyadic initiations with the parent. In turn, approximately 70% of the change in those child initiations came via a change in parental synchrony. Therefore, to the extent that the PACT intervention impacted these social communication skills it did so via a theoretically expected two‐step pathway: the first step being *within the dyad* from the increased parental synchrony causing greater child communication initiation and the second step being *within the child* from the improved communication initiation with the parent to improved autism symptom behaviours with the researcher at endpoint.

The 6‐year follow‐up of this PACT trial, which achieved an 80% follow‐up of the sample and preserved blinded assessment of the original treatment groups, evidenced a reduction of the original intervention effect on parental synchrony over time, but a sustained intervention effect on improved child dyadic initiations and the extent of autistic symptoms beyond the dyad (Pickles et al., 2016). Blinded teacher reports of adaptive behaviour skills also showed some evidence of a treatment effect. These sustained child improvements, after a maintained intention to treat analysis, are unique for such a long period after therapy and beg important questions as to what mediates them. Beyond longitudinal studies evidencing how early social communication skills predict later language outcomes (e.g. Adamson, Bakeman, Deckner, & Romski, 2009; Bottema‐Beutel, Yoder, Hochman, & Watson, 2014; Gulsrud et al., 2014), there is very limited research into the causal mechanism of downstream development, particularly in the context of interventions and their longer‐term impacts.

This current study aimed therefore to explore the mediation processes underlying these long‐term outcome results from the PACT trial. Considering our previous findings that endpoint symptom change in a research setting was strongly mediated by the change in midpoint child dyadic communication initiations with caregiver (Pickles et al., 2015), we hypothesised the same mediation through child initiations of the ADOS CSS change at 6‐year follow‐up. We further hypothesised that this would also be the case for the outcome teacher rated adaptive behaviour at 6 years. Furthermore, we explored the extent to which any mediation effects might be moderated by relevant baseline measures of child non‐verbal developmental ability [Age Equivalent (AE)], rigidity as reflected by insistence on sameness (IS) and early communication and symbolic behaviour skills (CSBS). Pre‐treatment AE and communication ability are commonly considered influences on treatment response (although a recent review of the literature (Trembath et al., 2019) found no overall evidence for this); we also postulated that IS might moderate mediation by affecting initial generalisation. So, for moderated mediation, we hypothesised that lower CSBS, lower non‐verbal AE and higher IS would be associated with less change in child initiations, and a weaker relationship between child initiations and the follow‐up outcomes.

## Methods

### Study design

The PACT trial (registered ISRCTN 58133827) was conducted in three specialist centres in the UK (London, Manchester and Newcastle) with 152 children with ‘core’ autism (ADOS symptom score > 12), aged 2 years to 4 years 11 months. A follow‐up study assessed 121(80%) of the trial participants. The median length of follow‐up from baseline to follow‐up was 82 months (IQR 78–85), and 69 months (IQR 65–71) from intervention endpoint to follow‐up. Participant flow is shown in Figure S1. Of the 77 children randomised to the PACT intervention, 59 (77%) were followed up together with 62 (83%) of the 75 participants randomised to receive treatment as usual. The mean age of the children at follow‐up was 10.5 years (*SD* 0.8). Assessment of primary outcomes was completed by assessors blind to intervention allocation. Table 1 shows descriptive statistics by intervention group at baseline and follow‐up. Full details of the trial and follow‐up study design including a CONSORT diagram have been reported previously (Green et al., 2010; Pickles et al., 2016). Summary statistics are shown in Table 1.

**Table 1:** Participant characteristics at baseline, trial endpoint and follow‐up by treatment group

	Baseline (*n* = 152)		Endpoint (*n* = 144)		Follow‐up (*n* = 121)	
	PACT (*n* = 77)	Treatment as usual (*n* = 75)	PACT (*n* = 74)	Treatment as usual (*n* = 70)	PACT (*n* = 59)	Treatment as usual (*n* = 62)
Sex						
Male	71 (92%)	67 (89%)	68 (92%)	62 (89%)	57 (97%)	54 (87%)
Female	6 (8%)	8 (11%)	6 (8%)	8 (11%)	2 (3%)	8 (13%)
Age (months)	44.7 (7.8)	45.0 (8.1)	58.0 (7.7)	58.2 (8.2)	127.3 (9.2)	127.2 (9.9)
Child Ini. (prop)^a^	0.23 (0.18)	0.24 (0.19)	0.35 (0.20)	0.26 (0.18)	0.30 (0.17)	0.27 (0.17)
CSS^b^	7.0 (1.4)	6.9 (1.9)	5.7 (1.7)	6.3 (1.6)	6.3 (1.9)	6.8 (1.8)
Vineland^c^	65.3 (8.1)	65.5 (9.0)	60.5 (15.3)	63.5 (15.0)	66.3 (21.3)	60.4 (16.6)
NV age‐eq^d^	27.0 (10.1)	25.3 (9.5)				
CSBS^e^	29.4 (7.2)	28.0 (9.0)				
RRB^f^	4.5 (3.8)	5.3 (4.0)				
Centre						
London	26 (34%)	26 (35%)				
Manchester	26 (34%)	26 (35%)				
Newcastle	25 (32%)	23 (31%)				

Data are mean (*SD*) or *n* (%). PACT, pre‐school autism communication trial.

^a^ Proportion of initiations among all child behaviours.

^b^ Comparative Severity Score.

^c^ Baseline Vineland parent rated, endpoint and follow‐up teacher rated.

^d^ Mullen non‐verbal age equivalent (months).

^e^ Communication and Symbolic Behaviour Schedule social composite raw score.

^f^ Insistence on sameness factor score from ADI‐restricted and repetitive items (Gotham et al., 2013).

### PACT intervention

The PACT intervention is a 1‐year developmental‐focused social communication intervention programme for young autistic children. Families in the active intervention group attended fortnightly, 2‐hr clinic sessions for 6 months, followed by 6 monthly booster sessions, and were asked to undertake 30 min of daily home practice between sessions. The theoretical background and underlying procedures in PACT therapy are detailed elsewhere (Aldred, Taylor, Wan, & Green, 2018; Green et al., 2010).

### Outcome measures

All measures were assessed, coded and rated blind to treatment allocation.

#### Autism behaviours—Autism Diagnostic Observation Schedule Calibrated Severity Score.^1^

The ADOS Calibrated Severity Score (ADOS CSS) combines social communication and sensory, restricted and repetitive behaviour ratings into an overall symptom score, which also allows comparison across different developmentally staged ADOS modules, essential for this long follow‐up study (Gotham, Pickles, & Lord, 2009). CSS has been shown to have high test–retest reliability (Janvier, Choi, Klein, Lord, & Kim, 2021). The ADOS CSS was calculated in this study for baseline, endpoint and follow‐up, at follow‐up, 43 children were assessed with module 1 (pre‐verbal/single words), 22 with module 2 (phrase speech) and 56 with module 3 (fluent speech). Scores range from 1 to 10 (1, 2 = minimal‐to‐no evidence of autism; 3, 4 = low; 5, 6, 7 = moderate; 8, 9, 10 = high severity). Reliability of ADOS CSS coding was assessed from 52 codings of 12 children assessed during the main trial and a further 50 codings of 25 children assessed at follow‐up, with overall ICC 0·73 (95% CI 0·58, 0·84).

#### Adaptive behaviour—Vineland Adaptive Behaviour Scales

We used the Vineland Adaptive Behaviour Scales composite (TVABS) standard scores (Sparrow, Cicchetti, & Balla, 2006) as rated by teachers. Teacher scores were available at the endpoint and follow‐up but were not included as an assessment at baseline; for this time point, we used parent report Vineland. As baseline assessment was conducted pre‐randomisation and teachers in middle childhood had had no involvement with families during the pre‐school treatment period, this measure is also effectively rated blind to treatment assignment. Parent and teacher TVABS are commonly highly correlated (e.g. Lane, Paynter, & Sharman, 2013; Szatmari, Archer, Fisman, & Streiner, 1994) although recent findings from our work suggest greater discrepancy (Moore et al., 2022).

### Mediators

#### Child initiations—Dyadic Communication Measure for Autism (DCMA)

The DCMA (Aldred, Green, & Adams, 2004; Green et al., 2010) involves blinded assessor real‐time coding of 8 min of a 12‐min video recording of free play between parent and child using a standard set of toys. Child communicative initiations (hereafter ‘Child Initiations’), defined as ‘verbal or non‐verbal communication acts used to be intentional or influence the responses of the other person’, are measured as a proportion of all child communication acts (see Appendix S1). This proportionality importantly increases the independence of parent and child codes during dyadic interaction. Researchers were trained to 75% inter‐rater reliability on 20 videos prior to coding and continued selected video coding for reliability maintenance during the coding process. The mean ICC estimation between coders across trial and follow‐up (Green et al., 2010; Pickles et al., 2016) was 0.70.

### Moderators assessed at baseline

#### Mullen Scales of Early Learning (Mullen, 1995)

We used the mean of the Visual Reception and Fine Motor subscales age equivalent scores as a measure of non‐verbal developmental ability (AE).

#### Communication and Symbolic Behaviour Scales—Developmental Profile (CSBS—Wetherby & Prizant, 2002)

We used the social composite raw scores from this parent questionnaire.

#### *‘Insistence on Sameness’ (IS) factor* (Gotham et al., 2013) from the Autism Diagnostic Interview‐Revised (ADI‐R; Lord, Rutter, & Le Couteur, 1994)

We used the validated IS factor, which consists of six ‘current’ items from this investigator‐based interview of the parent undertaken at baseline (Difficulty with Minor Changes in Routine, Compulsions/Rituals, Resistance to Trivial Changes in the Environment, Abnormal Response to Specific Sensory Stimuli, Sensitivity to Noise and Circumscribed Interests). The Cronbach's alpha of these items in our sample was 0.61, which was not considered to reduce their values as a predictor index for moderation.

### Statistical analysis

**Figure 1:**
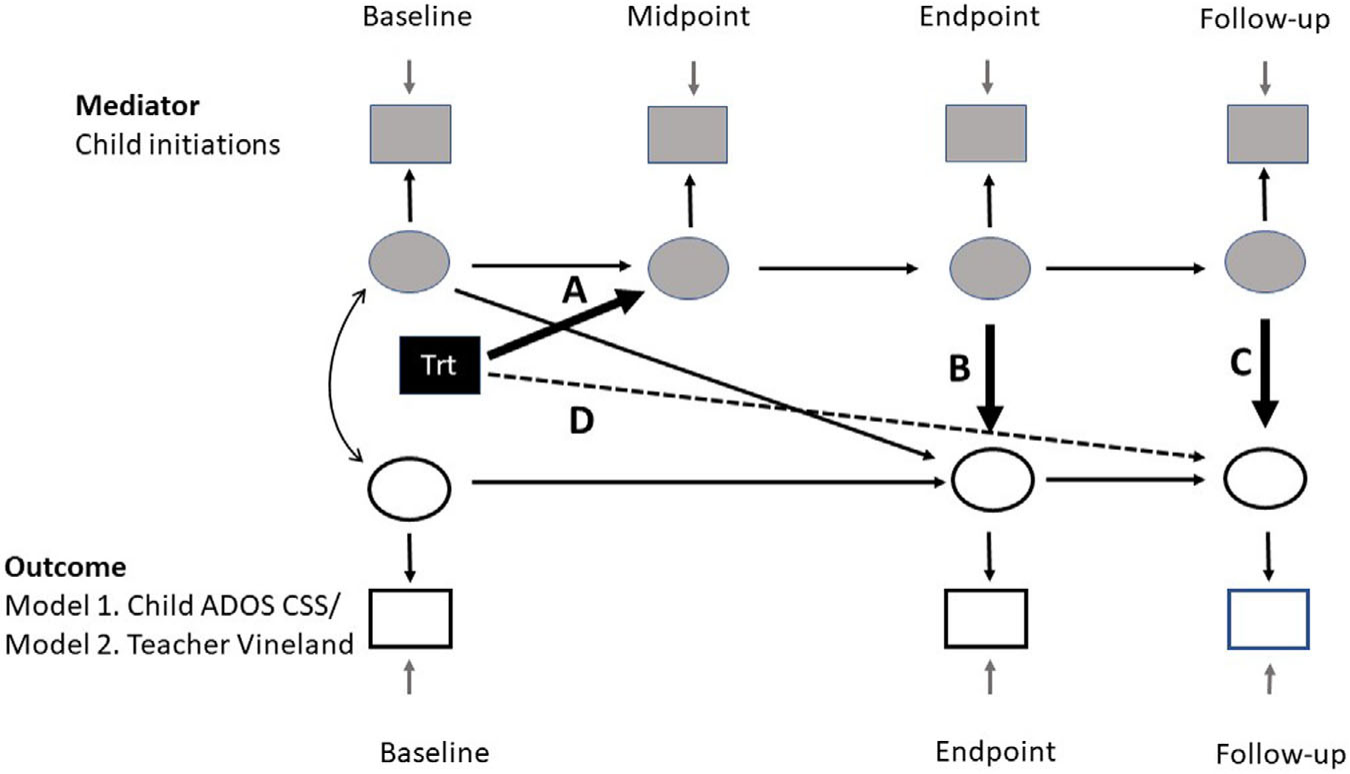
Parsimonious pre‐specified model of partial mediation of PACT therapy. Squares are observed variables, ovals are latent variables, double‐headed arrows indicate correlation and single‐headed arrows are direct effects. Single‐headed arrows lacking an origin indicate measurement errors. Dashed line D is the non‐mediated direct effect of treatment on the outcome at follow‐up, while paths A, B and C fall on paths of indirect effects between treatment and outcome at follow‐up [Color figure can be viewed at wileyonlinelibrary.com]

**Figure 2:**
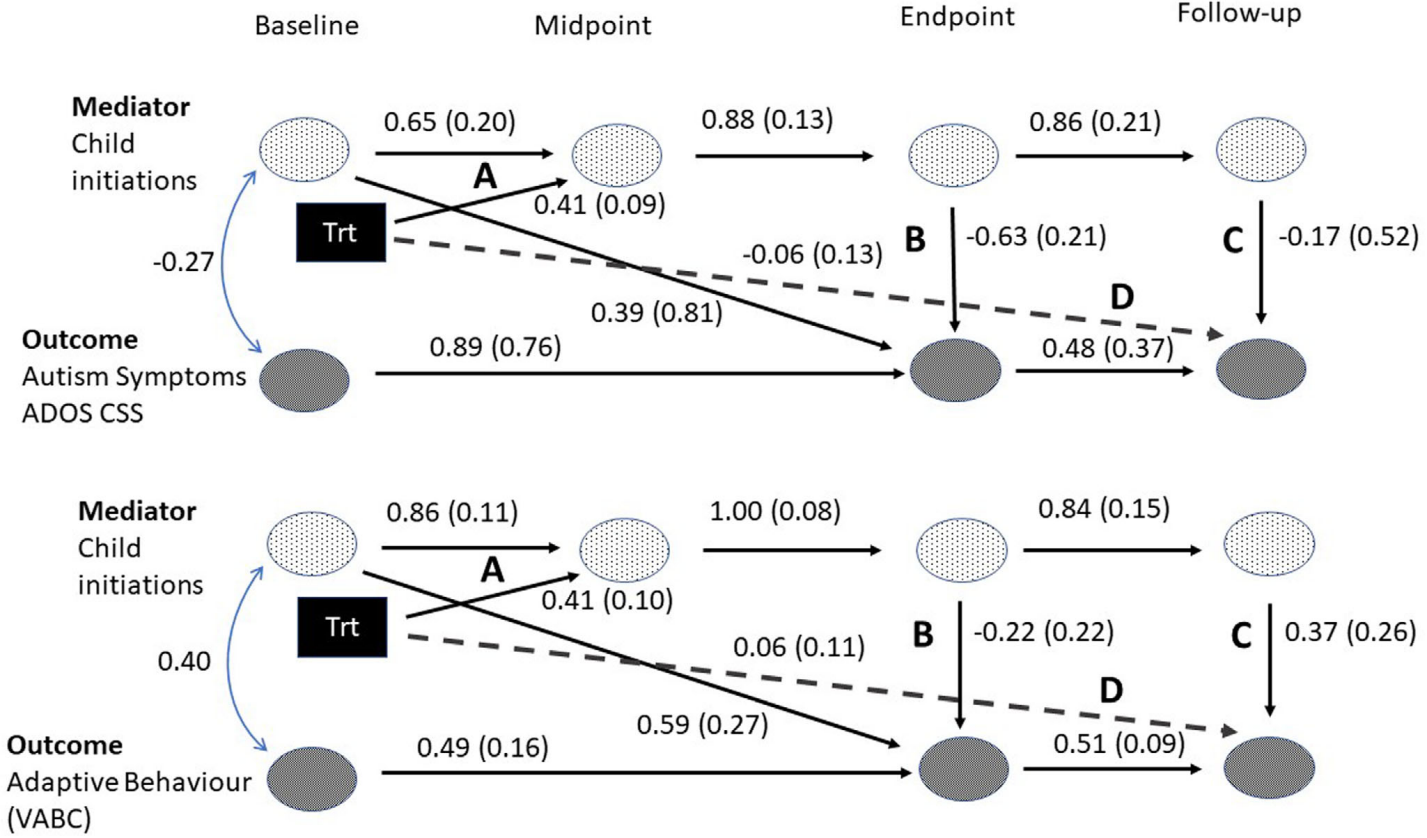
Standardised structural path coefficients (standard errors): top panel autism symptom behaviours (ADOS CSS), bottom panel adaptive functioning (VABC) (parent reported adaptive functioning at baseline, teacher reported adaptive functioning at endpoint and follow‐up). Squares are observed variables, ovals are latent variables, double‐headed arrows indicate correlation. and single‐headed arrows are direct effects. Dashed line D is the non‐mediated direct effect of treatment on the outcome at follow‐up, while paths A, B and C fall on paths of indirect effects between treatment and outcome at follow‐up. The coefficients for paths A and D from binary treatment a STDY, all others STDXY [Color figure can be viewed at wileyonlinelibrary.com]

This secondary analysis of the PACT trial data used structural equation modelling in Mplus 8.6 (Muthen & Muthen, 2017) as an approach to identifying mechanisms in longitudinal clinical trials (Goldsmith et al., 2018) which attempts to account for bias due to measurement error and baseline confounding. An analysis plan, including model specifications, was pre‐registered prior to any model fitting at https://osf.io/uxzws. In all models, the randomised intervention group was the predictor, and child initiations at treatment midpoint were a mediator of the treatment effect on the outcome at follow‐up. Baseline covariates were the randomisation stratification factors with paths added to baseline and follow‐up factors. A baseline mediator to trial outcome endpoint path was additionally included to reduce confounder bias (Landau, Emsley, & Dunn, 2018). The pre‐specified model illustrated complete mediation, with no treatment to outcome direct effect, and, for greater parsimony, interpretability and more stable estimation that reduced collinearity, only what were considered the essential mediational indirect paths. Developments from this pre‐registered model that arose from further consideration of the likely process and additions to minimise confounder bias are described in Appendix S2 and were undertaken during model construction rather than model fitting. The resulting more complex partial mediation pre‐specified model is shown in Figure 1, with the results displayed in Figure 2 and Table 2. In response to comments during peer review, we also undertook further post hoc exploratory analyses of 10 alternative mediational models. These are presented in Appendix S2, Figure S1 and Table S1. We consider these as a sensitivity analysis of the pre‐specified results.

**Table 2:** Effect estimates for Figure 1a for paths labelled A, B and C and selected indirect effects for ADOS Comparative Severity Score and teacher Vineland Adaptive Behaviour Composite outcomes

Effect	CSS outcome		TVABS outcome	
	Std estimate^a^, ^b^	95% CI^c^	Std estimate^a^	95% CI^c^
Individual paths				
Treatment to Mid‐initiation (A)	**0.41**	0.25 to 0.60	**0.41**	0.22 to 0.60
End‐initiation to End‐outcome (B)	**−0.63**	−1.03 to −0.25	−0.22	−0.74 to 0.15
FU‐initiation to FU‐outcome (C)	−0.17	−1.08 to 1.01	0.37	−0.07 to 0.95
Treatment to FU‐outcome (D)	−0.06	−0.29 to 0.19	0.06	−0.25 to 0.22
Indirect effects				
Treatment to End‐outcome	**−0.23**	−0.42 to −0.07	−0.09	−0.22 to 0.05
Treatment to FU‐initiations	**0.31**	0.19 to 0.53	**0.35**	0.22 to 0.59
Treatment to FU‐outcome	**−0.16**	−0.42 to −0.05	0.08	−0.07 to 0.41
Total effects				
Treatment on FU‐initiations	**0.31**	0.19 to 0.53	**0.35**	0.22 to 0.59
Treatment on FU‐outcome	**−0.22**	−0.44 to −.01	0.14	−0.02 to 0.27

^a^ STDY for Treatment effects and STDXY for mediators to outcomes.

^b^ Bold significant Wald *p* < .05 for unstandardised effects.

^c^ 2.5% and 97.5% bootstrap percentile estimates from 1000 replicates.

Models were estimated by maximum likelihood (ML or MLF) using MODEL INDIRECT for the estimation of mediated effects with 95% confidence intervals (CI) obtained using bootstrap (1,000 replicates). Treatment by moderator interaction effects on the midpoint mediator factor (Figure 1, path A) and endpoint outcome, and by treatment by mediator factor interaction on the paths from mediator to outcome (Figure 1, paths B and C), the latter specified using the XWITH command and numerical integration. Moderated paths also included the main effect of the moderator. We report delta‐method CI for mediated effects, with bootstrap being unavailable. Pre‐specified moderators examined were continuous measures for early child communication, insistence on sameness and non‐verbal developmental quotient.

Missing data patterns are reported in Appendix S3. No data points were excluded. The PACT trial enjoyed high levels of retention and thus a treatment of missing data under the assumption of Missing‐At‐Random, and thus ignorable under maximum likelihood, was reasonable. Tests of single coefficients are *p*‐values from Wald tests, and model comparisons involving multiple degrees of freedom are likelihood ratio chi‐square tests. *p*‐Values for indirect and total effects are from Wald tests of unstandardised effects. We report fully standardised (STDYX) estimates except for those for the binary treatment that are standardised only for outcome variability (STDY). In both cases, since the outcome is a continuous latent variable, standardisation is to this latent variable variability and not that of the indicator variable. Example scripts for the mediation and moderated mediation models are in Appendix S4.

Distributional assumptions for residuals from Mplus SEM are not easy to check. As a preliminary analysis, we examined normal probability plots for residuals for all response variables in the SEM, each taken in turn, from simple regressions that covaried for all the direct structural paths for that variable. For all midpoint, endpoint, and follow‐up variables, no departures from normality were evident. Some evidence of positive skew was evident for the baseline child initiations and CSS. However, no transformation was applied in order to retain a common scale over time. Our use of bootstrap CI provides some robustness to departure from normality.

### Ethical considerations

The PACT trial and follow‐up study were approved by the Central Manchester Multicentre Research Ethics Committee (05/Q1407/311). Written consent to participate was provided by at least one parent in each family enrolled in the study.

## Results

The CONSORT diagram (Appendix S5) and Table 1 show the high participant retention achieved until the formal endpoint of the trial and the success achieved in obtaining a follow‐up assessment after 6 further years. Table 1 shows descriptive statistics for the major study variables of DCMA proportion of child initiations (trial midpoint values not shown in the table were 0.40 (0.18) PACT and 0.28 (0.19) TAU), ADOS CSS and teacher TVABS standard score by treatment group, with their pairwise correlations shown in Table S1 and missing data patterns in Appendix S3.

We constructed a model in which trial baseline, midpoint (7 months), endpoint (13 months) and follow‐up (83 months) data formed a repeated measures mediational design where bias is minimised by using factors to account for any unreliability in the measurement of behavioural observed mediators which, uncorrected, can attenuate mediation estimates. Baseline measurements are included as potential confounders (Landau et al., 2018). Owing to randomisation, treatment is uncorrelated with baseline factors and other potential confounders.

After the test described in Appendix S2, the parsimonious model of partial mediation shown in Figure 1 was estimated for each of the two outcomes, with results shown in Figure 2 top and bottom panels respectively. Figure 2, Path A is the effect of the intensive period of treatment on child initiations (which it was hoped would persist to the trial endpoint during the maintenance support period of the trial and then beyond) through two indirect paths reflecting internalisation and even development of child patterns of behaviour. Figure 2, Path B is the effect of child initiation behaviour on contemporaneously assessed endpoint outcome (autism symptom behaviours or adaptive functioning). We expected some continuity in these symptoms to follow‐up. Figure 2, Path C is the effect of child initiations at follow‐up on outcome at follow‐up. Path D is an estimate of all other effects of treatment on follow‐up outcomes not mediated by the two mediation paths involving A and B, and A and C.

### Calibrated Severity Scores as outcome

The model of Figure 1 fitted well (χ^2^(27) = 31.08, *p* = .268, RMSEA = .032, CFI = 0.967).

The standardised factor loadings from baseline to follow‐up for child initiations were 0.56, 0.70, 0.60 and 0.53 and for CSS 0.53, 0.74 and 0.79. Standardised path coefficient estimates are shown in Figure 2, top panel.

Coded from brief videos, estimates of occasion‐specific variance (nominally measurement errors) for child initiations were substantial (51%–72% of total variance), but continuity between the ‘true‐score’ child initiations factors was high, with mid to endpoint standardised regression coefficient of 0.88 and endpoint to follow‐up 6 years later still 0.86. CSS factors showed high continuity over the relatively short duration of the trial (0.89) but quite modest continuity over the 6 years from trial endpoint to follow‐up (0.48).

As shown in Table 2, the estimated total effects at follow‐up of treatment increasing initiations (*p* = .001, CI 0.19 to 0.53) and reducing CSS symptom score (*p* = .041, CI −0.44 to −0.1) were significant. The treatment effect on child initiations seen at treatment midpoint persisted not just to the trial endpoint as previously reported (Green et al., 2010), but on to child initiations 6 years later at follow‐up (0.63, CI 0.38 to 1.10, *p* < .004, Pickles et al., 2016). The total indirect effect via child initiations of treatment transmitted to trial follow‐up CSS was close to significance (*p* = .057) and the total overall effect was significant (Wald *p* = .041, bootstrap CI −0.85 to 0.01). All three of the indirect effect estimates were significant, showing clear treatment effects on the follow‐up mediator, endpoint CSS and follow‐up CSS (viz, Figure 2, top panel: path calculus, 0.41*0.88*(−0.63*0.48 + 0.86*−0.17) = −0.16, CI −0.42 to −0.05). By contrast, the direct effect on endpoint CSS was small and non‐significant (path calculus, −0.16–0.06 = −0.22, *p* = .653). Overall therefore, the great majority (73%) of the treatment effect on follow‐up ADOS CSS occurred through the indirect path of increased child initiation at trial midpoint, with about one‐third of this effect on follow‐up arising from the persisting higher levels of initiation after the trial and two‐thirds from the effect on autism symptoms already observed by trial endpoint. Summary results from a set of models with alternative arrangements of mediational paths are shown in Table S2 and Appendix S2. None of these models gave any improvement in fit. The estimated total effect and child initiation‐mediated treatment effect showed great stability across all of these models.

### Vineland adaptive behaviour composite outcome

The model of Figure 1 was refitted with the Teacher VABS standard adaptive behaviour composite score as outcomes. It also fitted well (χ^2^(27) = 29.22, *p* = .350, RMSEA = .023, CFI = 0.991) with path coefficient estimates shown in Figure 2, bottom panel. The estimated factor loadings for the TVABS outcome were close to or at their upper limit of 1.00 but the estimated relationships between treatment and the mediator were similar to those from the CSS model. However, the relationship between child initiations and TVABS outcome is different, being non‐significantly negative at endpoint but significantly positive at follow‐up, and none of the indirect effects on the outcome is significant. The total effect on follow‐up was small but positive (ES = 0.14 on latent variable, bootstrap CI −0.02 to 0.27, Wald *p*‐value .052) and was divided almost equally (55% mediated) between direct (ES = 0.06, CI −0.25 to 0.22) and indirect effects (ES = 0.08, CI −0.07 to 0.41). This contrasted with the apparent effect by trial endpoint, which though non‐significant, was in the other direction (−0.09, CI 0.22 to 0.05). Summary results from the models with alternative arrangements of mediational paths are shown in Table S2 and Appendix S2. None of these models gave any improvement in fit, but the most complex gave a larger overall treatment effect estimate, and all gave rather lower estimates of indirect effects and correspondingly larger direct effects. Thus, while the evidence in these additional paths for child initiation mediation on TVABS now looks slightly less, the evidence for a total treatment effect is strengthened.

### Moderated mediation

We then examined the impact of three baseline moderators—non‐verbal ability (AE); social communication (CSBS) and insistence on sameness (IS)—on the four paths A, B, C and D shown in Figure 1 for each of the two outcomes; corresponding to six models, each testing four interactions. Like our baseline covariates, each moderator was allowed (hypothesis free) main effects onto the baseline and follow‐up mediators and outcomes. AE was analysed by median split (group means of 19 months and 33 months age equivalent against their chronological mean ages of 39 and 52 months, respectively, noting that due to the inclusion criteria of the trial, these represent ‘very low’ and ‘low’ age equivalents). At baseline, AE was positively associated with child initiations (*p* < .001) and Vineland (*p* < .001) and negatively associated with autism symptoms (*p* = .033). Additional effects on the follow‐up timepoint were not significant (CSS model, *p* = .192 for initiation, *p* = .133 for outcome; Vineland model *p* = .485 for initiation and *p* = .920 for outcome). While CSBS was positively associated with baseline Vineland, all other CSBS and IS main effects with baseline and follow‐up mediators and outcomes were non‐significant (*p*'s > .1).

The estimates and CI for the moderation effects are shown in Table 3. Only one of the 24 effects showed a bootstrap CI that excluded zero, that being for AE moderation of the path from follow‐up initiations to follow‐up autism symptoms (though the Wald test was not significant *p* = .064; all other moderation *p*'s > .1). The direction of effect was such that the association of higher levels of initiation going along with lower autism symptoms applied only in the higher AE group.

**Table 3:** Moderation effect estimates for Figure 1b for paths labelled A, B, C and D for Comparative Severity Score (CSS) and Teacher Vineland (TVABS)

Moderator	CSS outcome		Teacher vineland outcome	
	Std estimate^a^, ^b^	95% CI^c^	Std estimate^a^, ^b^	95% CI^c^
Non‐verbal age equivalence (AE)				
Treatment to mid‐initiation (A)	−0.09	−0.40 to 0.23	−0.17	−0.42 to 0.08
End‐initiation to end‐outcome (B)	0.08	−0.18 to 0.33	−0.16	−0.37 to 0.06
FU‐initiation to FU‐outcome (C)	**−0.39**	−0.69 to −0.09	0.04	−0.19 to 0.28
Treatment to FU‐outcome (D)	0.23	−0.19 to 0.65	0.17	−0.08 to 0.41
Social communication (CSBS)				
Treatment on mid‐initiations (A)	−0.03	−0.29 to 0.22	−0.02	−0.31 to 0.26
End‐initiation on end‐outcome (B)	−0.18	−0.42 to 0.07	0.12	−0.09 to 0.33
FU‐initiation on FU‐outcome (C)	−0.04	−0.33 to 0.25	0.10	−0.14 to 0.34
Treatment to FU‐outcome (D)	0.05	−0.24 to 0.35	0.03	−0.29 to 0.34
Insistence on Sameness (IS)				
Treatment on mid‐initiations (A)	−0.05	−0.41 to 0.31	−0.09	−0.36 to 0.18
End‐initiation on end‐outcome (B)	0.20	−0.04 to 0.44	−0.00	−0.20 to 0.20
FU‐initiation on FU‐outcome (C)	0.21	−0.05 to 0.46	0.02	−0.22 to 0.25
Treatment to FU‐outcome (D)	0.04	−0.34 to 0.43	0.13	−0.14 to 0.41

^a^ STDXY estimates.

^b^ Significant unstandardised coefficients (Wald *p* < .05) are in bold.

^c^ Delta method confidence 95% intervals.

## Discussion

The unusual length of follow‐up of the pre‐school PACT intervention (for 6 years into middle childhood), retaining the randomised trial groups with relatively little participant loss, allows modelling of the developmental processes underlying a sustained treatment effect that we believe is unique in the autism literature and indeed rare in psychosocial treatments for other conditions (but see Hektner, August, Bloomquist, Lee, & Klimes‐Dougan, 2014). The first hypothesis tested whether the sustained treatment effect on reduction in ADOS CSS symptoms would be mediated by treatment‐related improvement in child communication initiation in the parent–child dyad, as it had been to treatment endpoint. This hypothesis was supported. The great majority (73%) of the overall treatment effect on follow‐up CSS remains mediated through the increased child initiation at trial midpoint (itself mediated by improved parent synchrony, Pickles et al., 2015). The modelling in Figure 2 top panel indicates that this mediating path works through the strong initial increase in child initiations onto endpoint CSS and then mainly through the stability thereafter of that CSS change over the next 6 years. Additionally, however, the treatment‐induced change in child initiation is itself sustained through follow‐up; and while, at follow‐up assessment, the strength of the path between child initiation and CSS outcome is reduced (Figure 2, top panel, Paths B and C), the indirect effect of treatment on the child initiation mediator and the outcome remains significant, accounting for one‐third of the overall outcome effect (Table 2). As in our previous mediation analysis of endpoint outcome (Pickles et al., 2015), no direct effects of treatment or measured mediated pathways other than those through midpoint child initiations were found.

The second hypothesis tested whether these same improvements in midpoint child initiation would also mediate improved TVABS adaptive outcomes in school at follow‐up. This hypothesis received only partial support (Figure 2, bottom panel). As in the model for CSS, intervention increased child initiations and the continuity in these increased levels from midpoint to follow‐up was strong. However, higher levels of child initiation were not associated with higher TVABS during the period of the trial (although were suggestively so by follow‐up). Thus, by follow‐up, our hypothesised mediated path together with a direct effect of treatment gave a near‐significant small total positive treatment effect (Wald *p* = .052) on follow‐up TVABS.

Our overall interpretation is that the sustained reduction in CSS symptom score over 6 years from treatment endpoint after PACT intervention is caused by the intervention's initial impact on improving child dyadic initiation with the caregiver. The sustained child initiation change during the period from endpoint to follow‐up supports this longer‐term reduction and can perhaps best be understood as a *maintaining factor*, preserving improvements by mitigating the typically expected ‘washout’ trajectory (regression to mean) of treatment effects commonly observed in treatment studies (Morton & Torgerson, 2005). A further implication is that the ‘second stage’ generalisation process from the increased child's dyadic interaction with a parent to the reduced child autistic behaviours with a researcher (seen across restricted repetitive behaviours, sensory sensitivities as well as social communication ability; and which was identified in our previous mediation analysis to endpoint, Pickles et al., 2015), persists here, although somewhat attenuated, for six years into middle childhood. Regarding the TVABS outcome, our analysis shows a convincing effect of pre‐school PACT treatment on mid‐childhood school adaptation 6 years after treatment end (in itself an important finding), although the mediation of this effect through child initiations is less strong.

This demonstration of the mechanism of the sustained PACT treatment effect over such a long developmental period is consistent with the only other long‐term follow‐up in autism intervention science; the follow‐up to mean 8.8 years of 40 children after randomly allocated interventions pre‐school (Gulsrud et al., 2014). Although not an ITT analysis, this follow‐on assessment did show a relatively greater effect of joint attention intervention, compared to play‐ or behaviourally focused interventions, in improving 5‐year follow‐up growth trajectories in joint attention skills and language.

The established heterogeneity of ASD has led to an expectation that the varied profiles of an autistic individual's strengths and weaknesses may influence treatment response (Lord et al., 2021). However, consistent with Trembath et al. (2019), we found scant evidence for moderation effects, with only one of the 24 tests of moderation of mediated paths being significant. PACT delivery is designed to provide an individualised and developmentally adjusted intervention for child and family, and the absence of significant moderation on the path to the proximal target of child initiations suggests that the intervention as delivered in this sample is flexible enough to match the children's heterogeneous profiles of non‐verbal AE, CSBS and IS. With the possible exception of AE in middle childhood, we also found no evidence that these factors influenced the process of generalisation from child initiations with a parent to either autism symptoms or functioning at school.

The two outcome measures tested here represent different aspects of generalisation of intervention effect. The *ADOS CSS* is a measure of the autistic phenotype, including social communication abilities, restricted and repetitive behaviours and sensory sensitivities, undertaken in social interaction with a trained but unfamiliar adult. Its strengths lie in its strong psychometrics, construct validity in relation to the prototypical autism phenotype and predictive validity in relation to developmental outcomes (Gotham et al., 2009). In the PACT cohort, it showed more sensitivity to treatment effect than a BOSCC coding of the same tapes (Carruthers et al., 2021). The measure has recently been criticised (e.g. Timimi, Milton, Bovell, Kapp, & Russell, 2019) for embodying a ‘deficit‐based’ formulation of autism (in common with standard phenotypic and psychiatric nosology), but, to balance this, the assessment process is designed as a social setting that is sensitive to the child's behaviour and to provide multiple opportunities for showing both strengths and difficulties within autistic difference. In the PACT trial, the measure was valuable in allowing rigorous investigation of the generalisation of parent–child dyadic effects into a different context and with an unfamiliar adult; and the finding that the social communication focus of the PACT intervention results in improvements in both repetitive behaviour, sensory and the social communication domains (Pickles et al., 2016) is important evidence of cross‐domain generalisation of treatment outcomes (cf Sandbank et al., 2020). By contrast, the *teacher VABS* provides a challenging test of the generalisation of a treatment's effectiveness from clinic‐based intervention to adaptive function after many years in the naturalistic setting of school. Given this challenge, it is perhaps not surprising that treatment and mediation effects are less clearly identified for TVABS than the ADOS. However, there is still evidence at follow‐up of some mediation through child initiation, with an identified path and significant total effect between treatment and school adaptation in mid‐childhood. This finding is suggestive evidence in favour of the developmental model underlying the PACT therapy, whereby a therapy targeting a quite specific parental behaviours can result in a sequence of generalisations over time, leading to beneficial change spanning broader domains and different settings.

### Strengths and limitations

Mediational findings from randomised trials, with multiple repeated measures made blind to treatment assignment, provide a strong basis for causal inference, compared to cross‐sectional studies (Goldsmith et al., 2018). This study examined both autistic phenotype behaviours and a functional outcome, both of which are markedly independent of the direct intervention context and are measured blind (ADOS) or effectively blinded (TVABS). The change in raters, interactional partners and settings over time will have minimised bias due to correlated measurement error.

Our analyses took account of occasion‐specific measurement error in the mediator (which was relatively high in this sample), including baseline confounders, involved theoretically based moderators and were pre‐specified. Nonetheless, estimates for paths beyond the direct effect of randomised treatment may be biased due to omitted time‐varying confounders such as school and family environment. Thus, though we consider our findings to be more robust than any other study to date in identifying the mechanisms of treatment effects in autism, our estimates of mediated effects should nonetheless be treated with some caution. Consideration is also required in interpreting the one aspect of identified moderated mediation by AE; while our models controlled for the main effects of age group, our non‐verbal ability measure was an age‐equivalent one, chosen as being more inclusive and less subject to floor effects than standard scores, and some confounding of age and ability may have remained.

PACT is one of the largest trials in autism and is unique in having an extended 6‐year follow‐up period from the endpoint, with low levels of attrition allowing the intention to treat analysis at follow‐up. Nonetheless, the sample is modest by the standards of many other fields of medicine and larger studies would be desirable. Also, unlike many other trials in autism, the PACT sample comprised on average children with moderately low AE, and our findings may not necessarily generalise to those with uniformly higher levels of cognitive ability.

### Developmental and clinical implications

A feature of child dyadic communication initiation, as measured on DCMA, is that it reflects a child's *social motivation* and *engagement*, independent of verbal or other developmental ability. The proportionate coding method reduces non‐independence of measurement when rating dyadic interaction, allowing us to show a direction of causal effect from the caregiver's increased synchrony change to increased child communication initiation in the dyad (Pickles et al., 2015). The central role of social engagement in autistic development that we infer here is more widely accepted now but still runs counter to much past and current theory, for instance that autistic children are intrinsically socially avoidant. The developmental modelling in this current study provides a rigorous demonstration, on the contrary, of how central social motivation and engagement are for autistic children, how this is embedded in early social interactions in a way that is equivalent to that seen in neurotypical development, and the causal effects that this has then to influence later development for the autistic child, across context and through time. In this sense, autism is here re‐framed as a manifestation of individual difference (as neurodiversity) within the broader developmental science tradition, rather than a very separate developmental state (Green, 2022).

Clinically, these findings provide a demonstration of how a pre‐school intervention that produces focused improvement in child social communication can result in developmentally and adaptively meaningful downstream outcomes into middle childhood. This current analysis is the first to have demonstrated the mechanism of such effects. The findings support the logic model of the PACT intervention in its developmental targeting of child dyadic communication through therapy with parents. But the mechanistic analysis has further value in suggesting that other intervention models that succeed in increasing child communication initiations as we measure them here (an important caveat since apparently similar constructs measured differently may not be equivalent), could also have similar longer‐term outcome effects to PACT. This is an example of the generally important potential benefit of mechanistic analyses in clinical trials to identify ‘active processes’ of this kind and thus promote treatment evolution and innovation (Green, 2017; Marchette & Weisz, 2017).

## Supporting information


**Appendix S1.** Further detail on DCMA coding definitions and metrics.
**Appendix S2.** Further information on the statistical analysis.
**Appendix S3.** Missing value patterns in the outcome data.
**Appendix S4.** Example Mplus scripts.
**Appendix S5.** CONSORT diagram.
**Table S1.** Correlations among mediator and outcomes by treatment group.
**Table S2.** Comparison fit of extended models with additional mediational and control paths as numbered dashed paths in Figure S1.
**Figure S1.** Alternative mediation models: Dashed lines indicate additional paths explored in the models of Table S2.

## Footnotes

^1^
Our primary outcome in this study, the ADOS CSS, codes the nature and extent of a child's autism phenotypic behaviours observed during interaction with a trained assessor; described as ‘symptom severity’. Language in autism is evolving, and increasingly used outside the clinical context. Thus, we would like to note that we can understand ‘severity ’ in this context as ‘extent’ of autism behaviours rather than necessarily ‘worse’. We have emphasised this where appropriate in the text. The value of ADOS to our investigation is further discussed in the Discussion section of this paper.
