# Supplementary material for: Mediation of 6‐year mid‐childhood follow‐up outcomes after pre‐school social communication (PACT) therapy for autistic children: randomised controlled trial
Source: J Child Psychol Psychiatry. 2023 Apr 24;65(2):233–44. doi: 10.1111/jcpp.13798 (PMC10953331; doi:10.1111/jcpp.13798)

**Mediation of 6 year mid-childhood follow-up outcomes after pre-school social communication therapy for autistic children (PACT): randomised controlled trial**

Carruthers S, Pickles A, Charman T**,** McConachie H, Le Couteur A, Slonims, V., Howlin, P, Collum R, Salomone, E, Tobin H, Gammer I, Maxwell J, Aldred, C, Parr, J Leadbitter K, Green, J

**Supporting Information**

**Appendix S1. Further detail on DCMA coding definitions and metrics**

Child non-verbal or verbal communication acts which initiate interaction are defined as Child Initiations signalled by non-verbal behaviours, e.g. showing a toy to the adult, or pulling the adult's hand, and or by verbal behaviours e.g. saying, “get ball” or “some more”.

The child Communication Initiation is determined by the whole context; a Direct Attention may be distinguished from a Comment by the child’s positioning and non-verbal responses indicating the desire to direct the adult’s attention. A request for an object may be indicated by the child using a hand reach to a cup accompanied by a glance or a request for an action by the child lifting their arms up towards the adult. DCMA does not code behaviours as initiations which do not have a communicative function or intention, e.g. casting, banging, coughing, grasping, dumping. Communication acts which are difficult to code, e.g. singing, rhymes, jingles, repetitive speech, echolalic responses or stereotyped phrases/ scripts are coded as ‘other’. Inter-rater reliability estimates for child initiations were 0.59 during the trial and 0.80 during follow-up giving an averaged ICC for this analysis of 0.70.

**Appendix S2. Further information on the Statistical Analysis**

Model Justification**:** The modest inter-rater reliability of the DCMA child initiations ratings underscored the need to adjust for measurement error in the estimation of the mediated effect by the use of measurement models for the series of mediating variables, with mediation being examined through the series of factors. It is not uncommon for factors in such a series to be highly correlated giving rise to collinearity in their effects on the outcomes as well as negative estimates of the factor disturbances. With these concerns in mind our pre-specified model heavily restricted the potential mediational paths in order to deliver stable and interpretable estimates. Testing moderated mediation is also simplified if mediation can be shown as plausibly occurring on a single path, in our case the child initiations measure at trial midpoint. The main treatment had been delivered by the time of trial midpoint. We had therefore prespecified testing whether a path from treatment to trial endpoint was required in addition to that from treatment to trial midpoint. We tested this in conjunction with testing of the lagged path from midpoint mediator to endpoint outcome, as a single 2df test.

Following a reviewer’s concerns a series of 10 further models with additional mediational paths were estimated and are reported in Table S2. The most complex of these models failed to converge. None of the remainder offered a significant improvement in fit over our prespecified model, and several showed evidence of the collinearity we had feared, with absurdly large coefficients on one mediation path being countered by opposite effects on another. The problem of negative variance estimates for factor disturbances was avoided by parameterising as a quadratic term.

The choice of effects on the outcome from a lagged or contemporaneous mediator is an important but is rarely wholly unequivocal. Lagged effects have the appeal of a clear time ordering with change in the mediator preceding change in the outcome. However, where interventions operate on a mediator whose degree of persistence may vary, where the interval between assessments is long and whose effects on the outcome may take some time to accumulate (perhaps involving a generalization of behaviour that may depend upon both experience and maturational development) then covariance of the outcome with the contemporaneous mediator is more likely than with lagged mediator. In specifying our model we considered this likely to apply.

To avoid potential confounder mis-specification the initial models fitted differed from that pre-registered model by the inclusion of a covariance between the baseline child initiations and outcome factors and by a direct path from baseline mediator to outcome endpoint (Landau et al 2015). Additionally, baseline randomisation stratification factors were included as covariates whether significant or not, and were introduced as affecting baseline latent variables with their effects transmitted through autoregressive effects to mid- and endpoints that followed shortly after rather than as additional direct paths. Paths to follow-up were retained in view of the scope for additional effects during the extended follow-up period.

For the measurement models time invariant factor loadings and time constant measurement errors were specified. The estimated error variances from these models were bounded to be positive (except in the case of the single parent-report Vineland score at baseline where setting measurement error to zero made measured and factor variables equivalent) For the structural model a simplex structure with time-varying continuity coefficients and cross-lagged or cross-contemporaneous effects as shown in Figure 1. Goodness of fit is reported using chi-square, Root-Mean-Square-Error (RMSE and where values less than .05 reflect good fit).

We intended to undertake a sensitivity analysis by fitting a model that included the impact of possible covariance between outcomes that might arise from shared developmental change during the extended follow-up period but such a model proved to be not definitively identified. Figure S1 shows a base model with 6 additional numbered paths. Table S2 shows key estimates from several models that include various combinations of these additional paths. To achieve greater parsimony we initially tested whether we could remove the path from treatment to endpoint mediator shown as likely optional in the pre-specification (<https://osf.io/uxzws>) and from midpoint mediator to endpoint outcome. Likelihood ratio tests comparing the Base+1 and Base+1,4,6 models suggested these paths offered no improvement in model fit for neither the CSS outcome nor the TABC outcome (χ^2^(2) = 0.944, p=.624 and 2.005, p=.367 respectively). The Base+1 model of partial mediation was therefore used for the primary results reported in the main paper. A reviewer raised questions relating to the omission of other mediational paths. Though not pre-specified, the Table also presents results for a number of such alternative models. In all cases likelihood ratio tests against the partial mediation Base+1 model showed either no significant improvement in fit or the model could not be estimated. In the case of the CSS outcome, the estimates of mediational interest changed little. However, for the TABC outcome, where estimable, the estimates were much less stable with some models indicating very modest mediated effect but total effects were if anything larger.

**Appendix S3. Missing Value Patterns in the Outcome Data**

Missing-value patterns (1=complete 0=missing)

| Pattern

Percent | 1 2 3 4 5 6

71% | 1 1 1 1 1 1

16 | 1 1 1 1 0 0

7 | 1 1 1 1 1 0

1 | 1 0 0 0 0 0

<1 | 0 1 1 1 0 0

<1 | 1 0 0 0 1 0

<1 | 1 0 0 0 1 1

<1 | 1 0 1 0 0 0

<1 | 1 0 1 0 1 1

<1 | 1 1 0 0 0 0

<1 | 1 1 0 1 0 0

<1 | 1 1 1 0 0 0

100% |

Variables are

(1) initiation1 (2) adoseverity3 (3) initiation2 (4) initiation3 (5) adoseverity4 (6) initiation4: adoseverity1 complete

| Pattern

Percent | 1 2 3 4 5 6 7

62% | 1 1 1 1 1 1 1

14 | 1 1 1 1 1 0 0

7 | 1 1 1 1 1 1 0

6 | 1 1 1 1 1 0 1

3 | 1 1 1 1 0 1 1

1 | 1 1 0 0 0 0 0

1 | 1 1 1 0 0 0 0

1 | 1 1 1 1 0 0 0

<1 | 0 1 1 1 1 0 0

<1 | 1 0 1 1 0 0 0

<1 | 1 0 1 1 1 1 1

<1 | 1 1 0 0 0 1 1

<1 | 1 1 0 0 1 0 0

<1 | 1 1 0 0 1 1 0

<1 | 1 1 0 1 0 0 0

<1 | 1 1 1 0 0 1 1

100% |

Variables are (1) initiation1 (2) p_vinestd_base (3) initiation2 (4) initiation3 (5) t_vinestd_end (6) t_vinestd_fu (7) initiation4

**Appendix S4. Example Mplus Scripts**

TITLE: 7-11 Comparative Severity Score Mediation

Data:

File is mediation711.dat ;

Variable:

Names are

id proxytrt trt site d1 d2 d3 sex module agegrp age job2 paredu dq

insis csbs1 csbs3 pvine1 tvine3 tvine4 ini1 ini2 ini3 ini4 css1 css3

css4 severity;

Missing are all (-9999) ;

Usevariables are d2 d3 agegrp trt ini1 ini2 ini3 ini4 css1 css3 css4 ;

ANALYSIS: estimator is ML;

BOOTSTRAP = 1000;

MODEL:

! measurement model

FXb BY ini1@1 ;

FXm BY ini2@1 ;

FXe BY ini3@1 ;

FXf BY ini4@1 ;

CSb BY css1@1 ;

CSe BY css3@1 ;

CSf BY css4@1 ;

ini1 ini2 ini3 ini4 (a); !measurement errors equal (questionable for time4 )

css1 css3 css4 (b) ;

! structural model

FXb with trt@0 ;

CSb with trt@0 ;

FXb ON d2 d3 agegrp ;

CSb ON d2 d3 agegrp ;

FXf ON d2 d3 agegrp ;

CSf ON d2 d3 agegrp ;

FXm ON FXb trt ;

FXe ON FXm trt@0 ;

FXf ON FXe trt@0 ;

FXb (xb) ;

FXm (xm) ;

FXe (xe) ;

FXf (xf) ;

CSe ON CSb FXb FXm FXe trt@0 ;

CSf ON CSe FXb@0 FXf trt@0 ;

FXb WITH CSb;

CSb (cb) ;

CSe (ce) ;

CSf (cf) ;

MODEL CONSTRAINT:

NEW(xxb xxm xxe xxf ccb cce ccf bb) ;

xb = xxb*xxb;

xm = xxm*xxm;

xe = xxe*xxe;

xf = xxf*xxf;

cb = ccb*ccb;

ce = cce*cce;

cf = ccf*ccf;

b = bb*bb;

MODEL INDIRECT:

FXf IND trt;

CSf IND trt;

CSe IND trt;

OUTPUT: TECH1 stand CINT(BCbootstrap) sampstat residual ;

TITLE: PACT 7-11 CSS Outcome and DQ Moderated Mediation;

Data:

File is mediation711.dat ;

Variable:

Names are

id proxytrt trt site d1 d2 d3 sex module agegrp age job2 paredu dq_

insis csbs1 csbs3 pvine1 tvine3 tvine4 ini1 ini2 ini3 ini4 css1 css3

css4 severity;

Missing are all (-9999) ;

Usevariables are trt d2 d3 agegrp ini1 ini2 ini3 ini4 css1 css3 css4 dq dqtrt;

DEFINE:

dq=5+(dq_ - 100)/15 ;

dqtrt=dq*trt;

ANALYSIS: estimator is MLF;

type = random;

algorithm=integration;

MODEL:

! measurement model

FXb BY ini1@1 ;

FXm BY ini2@1 ;

FXe BY ini3@1 ;

FXf BY ini4@1 ;

CSb BY css1@1 ;

CSe BY css3@1 ;

CSf BY css4@1 ;

ini1 ini2 ini3 ini4 (a);

css1 css3 css4 (b) ;

!Structural Model

FXb ON d2 d3 agegrp dq ;

CSb ON d2 d3 agegrp dq ;

FXf ON d2 d3 agegrp dq ;

CSf ON d2 d3 agegrp dq ;

FXm ON FXb trt dq dqtrt;

FXe ON FXm Fxb ;

FXf ON FXe ;

FXb (xb) ;

FXm (xm) ;

FXe (xe) ;

FXf (xf) ;

CSe ON CSb Fxb FXe;

CSf ON CSe FXf trt dqtrt ;

FXb WITH CSb ;

CSb (cb) ;

CSe (ce) ;

CSf (cf) ;

!Interaction term and paths (main effect of IQ included above)

FXfdq | FXf XWITH dq;

CSf ON FXfdq ;

FXedq | FXe XWITH dq;

CSe ON FXedq ;

MODEL CONSTRAINT:

NEW(xxb xxm xxe xxf ccb cce ccf bb) ;

xb = xxb*xxb;

xm = xxm*xxm;

xe = xxe*xxe;

xf = xxf*xxf;

cb = ccb*ccb;

ce = cce*cce;

cf = ccf*ccf;

b = bb*bb;

OUTPUT: TECH1 stand cinterval sampstat residual ;

**Appendix S5. CONSORT Diagram:** Exclusion criteria, timeline of assessments, detail of PACT treatment and data completion. Times are given in months since baseline. PACT = Preschool Autism Communication Trial; TAU = treatment-as-usual.

Exclusion criteria:

children with a twin with autism; non-verbal age equivalent to 12 months or younger; epilepsy requiring medication; severe sensory impairment; severe mental illness in a parent; English not spoken between parent and child

Baseline assessments N=242

Randomisation N=152

**PACT intervention + TAU** N=77

1-year programme

1^st^ 6 months: 12 therapy sessions (2h)

2^nd^ 6 months: monthly support and extension sessions

Home practice: 20-30 min per day of planned practice activities with child

**TAU** N=75

Midpoint assessments (7 months)

Mediator N=146

Endpoint assessments (13 months)

N=146 CSS N=138 Vineland

Follow-up assessments

(82 months)

N=121 CSS, N=112 Vineland

assessments (13 months)

N=146 CSS N=138 Vineland

SEM Mediation Analysis

N=152 in all SEM analyses except N=147 with CSBS moderation

**Table S1**. **Correlations among mediator and outcomes by treatment group**

**Treatment as Usual**

| Variables | (1) | (2) | (3) | (4) | (5) | (6) | (7) | (8) | (9) | (10) |
| --- | --- | --- | --- | --- | --- | --- | --- | --- | --- | --- |
| (1) DCMA initiation1 | 1.00 |  |  |  |  |  |  |  |  |  |
| (2) DCMA initiation2 | 0.23 | 1.00 |  |  |  |  |  |  |  |  |
| (3) DCMA initiation3 | 0.15 | 0.32 | 1.00 |  |  |  |  |  |  |  |
| (4) DCMA initiation4 | 0.32 | 0.23 | 0.32 | 1.00 |  |  |  |  |  |  |
| (5) ADOS CSS1 | -0.13 | -0.02 | 0.00 | -0.20 | 1.00 |  |  |  |  |  |
| (6) ADOS CSS3 | -0.05 | -0.25 | -0.27 | -0.30 | 0.35 | 1.00 |  |  |  |  |
| (7) ADOS CSS4 | -0.08 | -0.18 | -0.07 | -0.27 | 0.15 | 0.22 | 1.00 |  |  |  |
| (8) Parent Vineland 1 | 0.05 | 0.14 | 0.27 | 0.20 | -0.04 | -0.06 | -0.11 | 1.00 |  |  |
| (9) Teacher Vineland 3 | 0.21 | 0.21 | 0.29 | 0.33 | -0.15 | -0.34 | -0.13 | 0.63 | 1.00 |  |
| (10) Teacher Vineland 4 | 0.19 | 0.26 | 0.41 | 0.39 | -0.23 | -0.49 | -0.28 | 0.47 | 0.68 | 1.00 |
|  | | | | | | | | | | |

**PACT**

| Variables | (1) | (2) | (3) | (4) | (5) | (6) | (7) | (8) | (9) | (10) |
| --- | --- | --- | --- | --- | --- | --- | --- | --- | --- | --- |
| (1) DCMA initiation1 | 1.00 |  |  |  |  |  |  |  |  |  |
| (2) DCMA initiation2 | 0.23 | 1.00 |  |  |  |  |  |  |  |  |
| (3) DCMA initiation3 | 0.15 | 0.32 | 1.00 |  |  |  |  |  |  |  |
| (4) DCMA initiation4 | 0.32 | 0.23 | 0.32 | 1.00 |  |  |  |  |  |  |
| (5) ADOS CSS1 | -0.13 | -0.02 | 0.00 | -0.20 | 1.00 |  |  |  |  |  |
| (6) ADOS CSS3 | -0.05 | -0.25 | -0.27 | -0.30 | 0.35 | 1.00 |  |  |  |  |
| (7) ADOS CSS4 | -0.08 | -0.18 | -0.07 | -0.27 | 0.15 | 0.22 | 1.00 |  |  |  |
| (8) Parent Vineland 1 | 0.05 | 0.14 | 0.27 | 0.20 | -0.04 | -0.06 | -0.11 | 1.00 |  |  |
| (9) Teacher Vineland 3 | 0.21 | 0.21 | 0.29 | 0.33 | -0.15 | -0.34 | -0.13 | 0.63 | 1.00 |  |
| (10) Teacher Vineland 4 | 0.19 | 0.26 | 0.41 | 0.39 | -0.23 | -0.49 | -0.28 | 0.47 | 0.68 | 1.00 |
|  | | | | | | | | | | |

**Table S2.** **Comparison Fit of Extended Models with Additional Mediational and Control Paths as numbered dashed paths in Figure S1.**

| Model | df | CSS outcome GoF Chi-square | Total  Effect | Indirect  Effect | T-VABS outcome  GoF Chi-square | Total  Effect | Indirect Effect |
| --- | --- | --- | --- | --- | --- | --- | --- |
| Base (full mediation) | 28 | 31.323 | -0.36 | -0.36 | 29.667 | 0.12 | 0.12 |
| Base+1 (partial mediation) | 27 | 31.078 | -0.22 | -0.16 | 29.220 | 0.14 | 0.08 |
| Base+1,4,6 | 25 | 30.134 | -0.22 | -0.16 | 27.215 | 0.15 | 0.04 |
| Base+1,2 | 26 | 30.368 | -0.22 | -0.15 | 27.745 | 0.14 | 0.02 |
| Base+1,2,3 | 25 | 30.277 | -0.22 | -0.14 | No convergence |  |  |
| Base+1,2,3,4 | 24 | 30.214 | -0.22 | -0.14 | 26.750 | 0.29 | 0.03 |
| Base+1,2,3,4,5 | 23 | No convergence |  |  | No convergence |  |  |

**Figure S1. Alternative Mediation Models: Dashed lines indicate additional paths explored in the models of Table S2.**
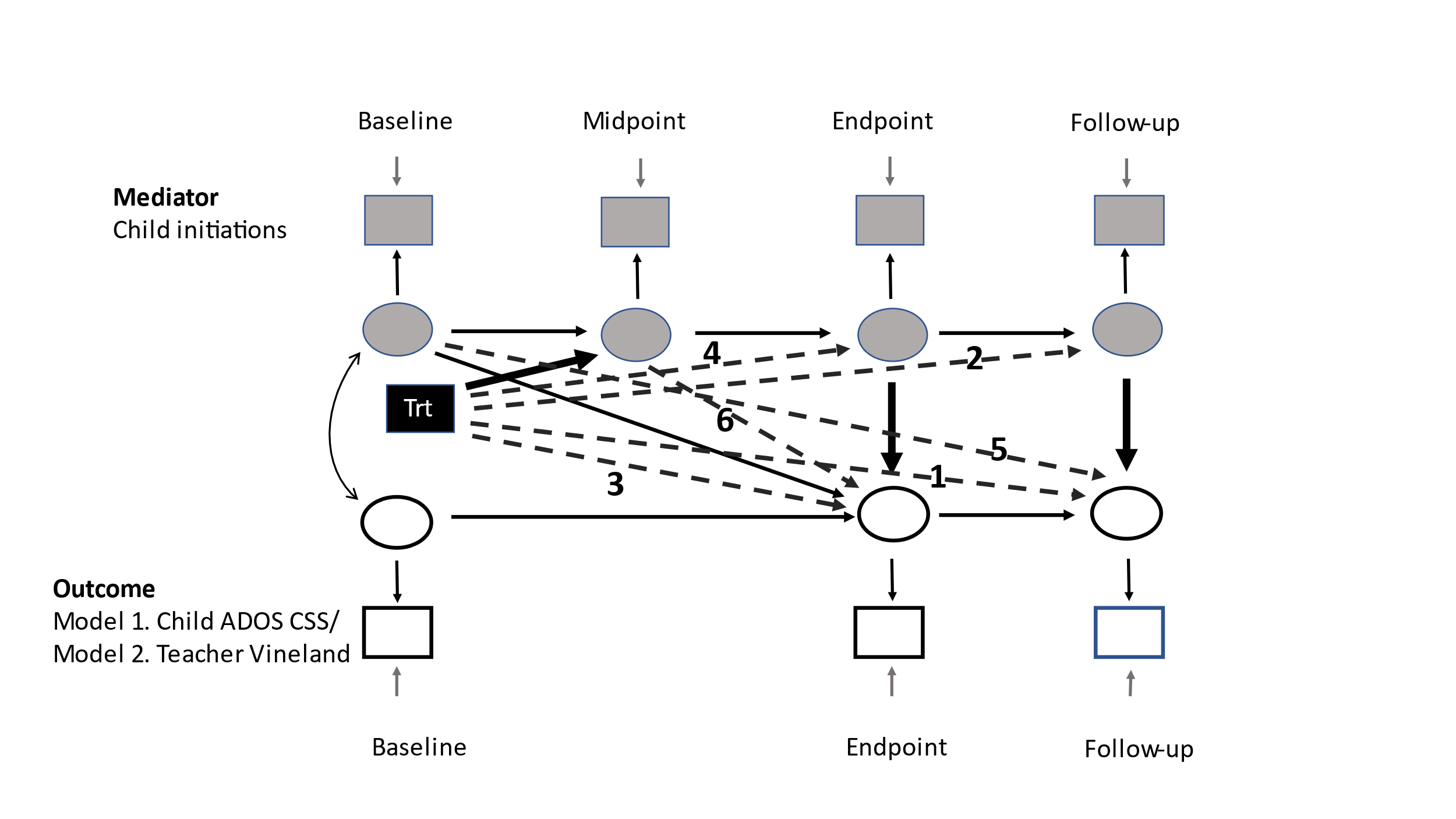

Supplement: Supplementary file 1 — Appendix S1. Further detail on DCMA coding definitions and metrics. Appendix S2. Further information on the statistical analysis. Appendix S3. Missing value patterns in the outcome data. Appendix S4. Example Mplus scripts. Appendix S5. CONSORT diagram. Table S1. Correlations among mediator and outcomes by treatment group. Table S2. Comparison fit of extended models with additional mediational and control paths as numbered dashed paths in Figure S1. Figure S1. Alternative mediation models: Dashed lines indicate additional paths explored in the models of Table S2. [file JCPP-65-233-s001.docx]
